# The Effect of Massage Therapy by Patients’ Companions on Severity of Pain in the Patients Undergoing Post Coronary Artery Bypass Graft Surgery: A Single-Blind Randomized Clinical Trial

**Published:** 2014-07

**Authors:** Sied Saeed Najafi, Fazlola Rast, Marzieh Momennasab, Mahmood Ghazinoor, Fereshteh Dehghanrad, Sied Ali Mousavizadeh

**Affiliations:** 1 Department of Medical Surgical Nursing, School of Nursing and Midwifery, Shiraz University of Medical Sciences, Shiraz, Iran;; 2 Department of Cardiac Surgery, Nemazee Teaching Hospital , School of Medicine, Shiraz University of Medical Sciences, Shiraz, Iran;; 3 Social Determinants Health Research Center, Yasuj University of Medical Sciences, Yasuj, Iran

**Keywords:** Massage Therapy, Companion, Pain, Coronary Artery Bypass Graft Surger

## Abstract

**Background:** Pain on mid sternotomy incision site after Coronary Artery Bypass  Graft  Surgery (CABG) is a common problem that causes sleep disturbance, delayed wound healing, and increased use of analgesic drugs. Massage therapy which is mostly performed by healthcare providers is a non-pharmacological approach for managing this pain. The present study aimed to determine the effect of massage therapy by patient’s companion on the severity of pain in post CABG patients.

**Methods: **In this randomized single-blind clinical trial, 70 post CABG patients were randomly divided into an intervention and a control group. The intervention group received massage by one of their relatives who was trained by an expert nurse. The control group, on the other hand, received routine care. The pain intensity was assessed by Visual Analogue Scale (VAS) before and immediately, 30, 60, and 120 minutes after the intervention. Then, the data were entered into the SPSS statistical software (version 16) and analyzed using repeated measures ANOVA and post-hoc test (Scheffe).

**Results:** At the beginning of the study, no significant difference was found between the two groups regarding the pain severity. In the intervention group, the pain severity significantly decreased in all the four time points after the intervention (P=0.001). However, no significant difference was observed in this regard in the control group.

**Conclusion: **Massage therapy by patient’s companion trained by a nurse was an effective strategy for pain management in post CABG patients. This could also promote the patient’s family participation in the process of care.

**Trial Registration Number: **IRCT201208218505N3.

## Introduction

Coronary Vascular Diseases (CVD) are the most prevalent heart diseases that are mostly treated using non-invasive methods. Although these therapeutic methods are effective, many sufferers may need vascular repair. Coronary Artery Bypass Graft surgery (CABGs) is one of the common and effective treatments for reduction or removal of cardiac angina.^1^ Considering the prevalent application of this therapeutic method throughout the world, we could claim that CABG provides an opportunity to increase the quality of life of thousands of patients every year.^2^

CABG is sometimes accompanied by some complications and problems, especially deep visceral and continuous pains that usually continue until two days after the surgery and then will be reduced gradually. These pains can delay the recovery and cause patient dissatisfaction.^3^^-^^5^ In spite of the noticeable growth of technology, musculoskeletal pain has remained as one of the psycho-physical health problems because it cannot be completely relieved by medicine.^6^ At present, narcotic sedatives are being used to relieve the pain, but they can lead to respiratory suppression and wide and non-preventable painlessness. Although using narcotic drugs in the patients who undergo heart surgery is necessary primarily, application of large amounts of such drugs delays the recovery and also increases the length of hospital stay.^7^

By recognition of risky patients and prevention of the side-effects, nurses play important roles in control of pain.^8^ Therefore, finding new methods to deal with such challenges is necessary for these patients.^9^ Massage therapy is accounted to be one of the methods of complementary medicine as well as one of the oldest methods of healthcare. The origin of massage therapy has been found in old texts of ancient China as well as in Hippocrates’ writings.^10^ Massage therapy was considerably spread during the last two decades. Studies have shown that massage therapy alone or accompanied by other complementary treatments was beneficial in reduction of pain and psychological distress.^11^ Massage has been used to reduce challenges, such as pain and stress which are the causes of anxiety in hospitalized patients.^12^^,^^13^ Yet, contradictory results have been obtained regarding the effect of massage therapy on pain. For instance, the results of a research showed that foot massage did not have any significant effects on pain among the patients undergoing heart surgery.^14^ Another research, however, reported the positive effect of foot reflexive massage on the sternotomy site on the severity of pain after CABGs.^15^ Therefore, more studies are required to be conducted on the effect of massage therapy in the patients undergoing cardiovascular surgery.^16^

Up to now, several studies have emphasized the effect of massage on reduction of pain and anxiety in cardiac patients.^6^^,^^16^^,^^17^ However, patients’ companions did not have any roles in doing the massage in any of the previous studies. Also, no studies have been performed on the effect of massage therapy by patients’ companions on the severity of pain caused by sternotomy after CABGs. Participation of relatives in the process of care has positive effects on the length of hospital stay as well as provision of care.^18^ The presence of family also constitutes an important source of support for better recovery.^19^^,^^20^ Of course, this will be effective if relatives are adequately informed about the patients’ conditions and appropriately trained by the medical and nursing staff.^21^ The present study aims to determine the effect of massage therapy by patients’ companions on the severity of sternotomy site pain in the patients undergoing CABGs.

## Materials and Methods

This randomized, single-blind, controlled trial was conducted on the patients hospitalized in open heart surgery wards of Nemazee and Shahid Faghihy teaching hospitals, Shiraz, Iran between November 2012 and March 2013. Considering the power of 0.8 and Altman monogram, a 70- subject sample size was determined for the study. Nevertheless, considering the probable loss during the study, 78 eligible patients were enrolled into the study and divided into two groups. Among these patients, 6 were not willing to participate in the study and 4 discontinued their participation due to severe pain and need for analgesic drugs.

Based on consultation with the patients’ physician the day before the operation, the researcher recognized if the patients met the inclusion criteria and invited them to take part in the research. The inclusion criteria of the study were being candidate for CABGs, being willing to participate in the research, being 18-70 years old, being hospitalized for at least 3 days after the operation, being oriented to time, place, and person, not having used narcotics and alcoholic drinks during the last two months, not having the history of nervous, neurovascular, psychiatric, and respiratory disturbances, and not suffering from coagulation disorders. On the other hand, the exclusion criteria of the study were reduction of level of consciousness, instability in hemodynamic status, unwillingness to continue cooperation, presence of coagulation problems (according to the natural limit of INR,PT), increase in the period of connection to pump to more than four hours, and suffering from chronic and malignant pain. The subjects were selected using convenient sampling and those who met the inclusion criteria were allocated to the intervention or control groups through block randomization. At first, the objectives of the research were explained to the patients and written informed consents were obtained. On the third day after the operation, the patients were examined by their physician and, in case of confirmation, were included in the study. 

**Figure 1:**
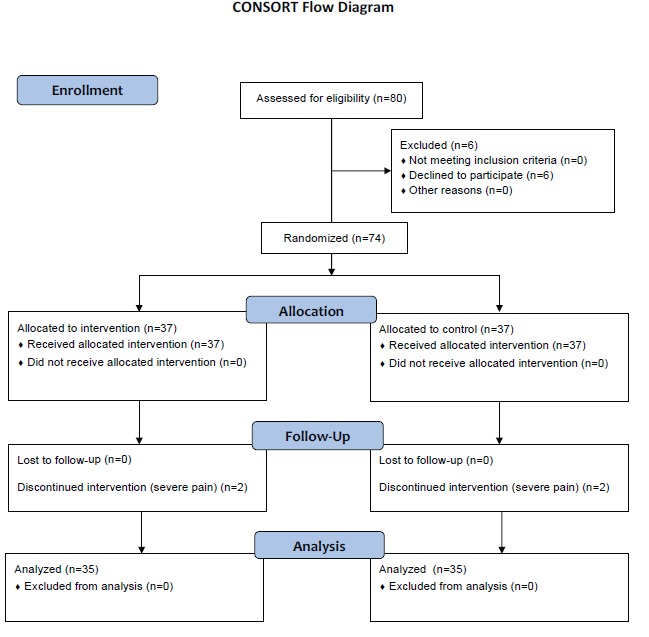
Design and protocol of the study.

In the intervention group, one of the patients’ relatives who had active attendance at the time of hospitalization was selected and invited. The patients’ companions were then trained regarding massage therapy through face-to-face discussion, an educational CD, and practice by simulation. It should be noted that the training period varied from 60 to 90 minutes according to the companions’ learning abilities. After all, the participants’ competency in massage therapy was approved by a trained nurse. The intervention was performed when the patients were transferred from ICU to cardiac surgery ward on the third day after the operation. The patients who had severe pain and needed analgesics were excluded from the study. The study patients’ demographic characteristics were recorded in a data recording form. Thereafter, their severity of pain was assessed using McGill’s Visual Analogue Scale (VAS) whose validity and reliability have been confirmed previously. VAS is a beneficial tool to evaluate patients’ pain after surgery.^22^ This instrument is like a ruler numerated from zero to ten. Accordingly, scores of 1-3, 4-6, 7-9, and 10 represent mild, moderate, severe, and intolerable pain, respectively. The instruction for use of VAS was explained to the patients on the first day of hospitalization. In the intervention group, massage therapy was carried out by Thailand classic method by patients’ companions under the nurse’s supervision in a private room for 30 minutes. The procedure was carried out by soaking hands with sweet almond oil which is the most common oil applied in massage therapy^23^ and massage was done at back, lumber, shoulders, arms, forearms, the palm and fingers of both hands, thigh, foreleg (except for donor places), soles, insteps and fingers of feet, abdomen, and neck muscles according to the patients’ tolerance. The severity of the patients’ pain was reassessed and recorded immediately, 30 minutes, 60 minutes, and two hours after the intervention. The control group, however, received the routine care on the third day of operation and their pain scores were recorded at the same time intervals as the intervention group. Of course, the nurse and the patients’ companions were present at the patients’ bedsides for 30 minutes to provide similar conditions to the intervention group. It should be mentioned that pain was assessed by a researcher who had no information about the study groups. In addition, the ward’s routine care was performed for both groups (figure 1). The intervention group patients’ satisfaction from massage therapy was assessed using a 5-point Likert scale ranging from very low to very much.

After all, the data were entered into the SPSS statistical software (V. 16) and analyzed using independent t-test, paired t-test, and repeated measures Analysis of Variance (ANOVA) with schefe post hoc test. Besides, P<0.05 was considered as statistically significant.

*Ethical Considerations*

This study was approved by the Research Vice-chancellor and Ethics Committee of Shiraz University of Medical Sciences (Ct-6260(. All the research samples signed written informed consents and were ascertained that their individual information would remain confidential and their participating in the study was voluntary.

## Results

Out of the 70 participants, 38 (54.3%) were male and 32 (45.7%) were female. The mean age of the study samples was 59.77±7.28 years and 91.4% (n=64) of them were married. The mean age of the patients in the intervention and control groups was 59.28±7.11 and 60.25±7.52 years, respectively. Most of the participants’ companions in the research (68.6%) were the patients’ children and the remaining were other relatives. At the beginning of the study, no significant difference was observed between the two groups regarding the demographic characteristics, including age, sex, level of education, and marital status (table 1). In addition, the mean and standard error of the severity of pain was 6.56±1.74 and 7.11±1.82 in the intervention and the control group, respectively, and the difference was not statistically significant. However, the results of paired t-test showed a significant reduction in the intervention group’s mean score of pain immediately, 30, 60, and 120 minutes after the intervention compared to before the intervention (P=0.001). The highest reduction in pain was observed 60 minutes after the intervention. On the other hand, no significant difference was observed between the control group’s mean scores of pain before and after the intervention. The results of repeated measures ANOVA showed that the severity of pain among the intervention group participants who received massage by their companions was significantly lower compared to the control group (P=0.001). Mean and standard deviation of pain severity in the two groups at various time intervals and the difference between the two groups have been presented in table 2.

**Table 1:** Demographic characteristics of the patients in the intervention and the control group

**Variables**	**Characteristics**	**Groups**		**P value**
		**Intervention** **No. (%)**	**Control** **No. (%)**	
Sex	Male	19 (54.3)	19 (54.3)	0.860
	Female	16 (45.7)	16 (45.7)	
Marital status	Married	32 (91.4)	32 (91.4)	0.754
	Single or widowed	3 (8.6)	3 (8.6)	
Level of education	Illiterate	14 (40)	16 (37.1)	0.215
	Elementary and secondary	20 (57.1)	16 (37.1)	
	High school or above	1 (2.9)	3 (8.8)	

**Table 2:** Comparison of pain intensity in the 5 time intervals in the two groups (mean±SD)

**Groups** **Period**	**Before the intervention**	**Immediately after the intervention**	**30 minutes after the intervention**	**60 minutes after the intervention**	**120 minutes after the intervention**
intervention	6.56±1.74	3.41±1.77	3.01±1.78	2.82±1.83	3.25±1.91
Control	7.11±1.82	7.07±1.81	7.09±1.81	7.13±1.71	7.17±1.71

P-Repeated measures ANOVA for main effect of intervention and time is statistically significant (P=0.001)

All the patients in the intervention group were satisfied with massage therapy; such a way that 60.0% and 40.0% of these patients described their satisfaction rate as “very much” and “much”, respectively. 

## Discussion

The results of this study showed that offering the patients’ companions to participate in taking care of the patient after being sufficiently trained regarding massage therapy was effective in reducing the severity of pain in post CABG patients. This finding has been supported by other researches, as well. Some studies conducted on heart surgery patients have indicated the significant effect of massage therapy on reduction of stress, anxiety, and pain.^6^^,^^16^^,^^17^ In another study, massage therapy was performed on 53 inpatients at medical, surgical, and obstetric wards for a period of 30 minutes in one session or more. The results showed that the intervention was effective in reduction of pain and improvement of quality of sleep, tranquility, recovery period, and healing process.^24^ Also, numerous studies on massage therapy in cancer patients have demonstrated its significant effects on perceived stress, quality of life, and common symptoms, such as pain, nausea, anxiety, mood disturbance, fatigue, and disturbed sleep. A large study on 1,290 patients revealed that a single massage reduced the symptoms levels by 21-52 %.^25^

Moreover, several studies have shown the significant effects of patients’ companions’ participation in care process on the quality of life and the related indexes in hospitalized patients.^26^^,^^27^ The results of two studies carried out in this area reported that massage therapy by companions as well as nurses was effective in reduction of anxiety, systolic blood pressure, heart rate, and respiratory rate among the male patients hospitalized in CCU.^28^^,^^29^ The results of another study indicated that doing acquainted sensory stimulations by patients’ companions during the first six days of admission of patients with brain trauma at ICU increased the level of consciousness compared to the control group.^27^ Also, use of organized sensory stimulation by family members led to a significant increase in the level of consciousness and cognitive behavior state in brain injured patients.^30^

The effect of interpersonal relationships as a contributing factor to the benefits of massage should also be considered. After experiencing a major surgery and a stressful situation in a surgical ward, patients can benefit from the attention of a family member providing support and comfort.^31^ The findings of the current study showed that participation of the patients’ relatives in the care process eliminated the pain, eventually increasing the patients’ and companions’ satisfaction. According to the results of the previous studies, the active support of relatives in the treatment process is highly valuable.^32^ In our socio-cultural context, familial relationships influence the whole dimensions of life of individuals, especially in illness and crisis.^33^

Furthermore, involvement of patients’ companions as family caregivers in massage therapy can lead to continuation of this effective intervention after discharging from the hospital. Of course, appropriate education and training must be considered by nurses with respect to patient safety and provision of effective care.^34^

## Conclusion

This study revealed new information about the effectiveness of massage therapy by patients’ companions that could be observed by the nursing personnel in order to train the companions and recommend such treatments to patients. The study results showed that massage therapy could be safely and effectively delivered by the companions in cardiothoracic surgical ward, leading to significant reductions in pain and patient satisfaction. Yet, further studies are recommended to assess the effect of massage therapy on these patients’ severity of pain in longer time periods.
